# Use of animal models for exome prioritization of rare disease genes

**DOI:** 10.1186/1750-1172-9-S1-O19

**Published:** 2014-11-11

**Authors:** Damian Smedley, Sebastian Kohler, William Bone, Anika Oellrich, Jules Jacobsen, Sanger Mouse Genetics Project, Kai Wang, Chris Mungall, Nicole Washington, Sebastian Bauer, Dominic Seelow, Peter Krawitz, Cornelius Boerkel, Christian Gilissen, Melissa Haendel, Suzanna E Lewis, Peter N Robinson

**Affiliations:** 1 Mouse Genetics Project, Wellcome Trust Sanger Institute, Hinxton, UK; 2 Institute for Medical and Human Genetics, Charite-Universitatsmedizin Berlin, Augustenburger Platz 1, 13353 Berlin, Germany; 3 Max Planck Institute for Molecular Genetics, Ihnestr. 63–73, 14195 Berlin, Germany; 4 National Institutes of Health, NHGRI, UDP Translational Laboratory, Rockville, USA; 5 Center for Applied Genomics, Childrens Hospital of Philadelphia, University of Pennsylvania, Philadelphia, PA 19104, USA; 6 Genomics Division, Lawrence Berkeley National Laboratory, Berkeley, CA 94720, USA; 7 Department of Neuropaediatrics Charite-Universitatsmedizin Berlin, Augustenburger Platz 1, 13353 Berlin, Germany; 8 Berlin Brandenburg Center for Regenerative Therapies, Charite-Universitatsmedizin Berlin, Augustenburger Platz 1, 13353 Berlin, Germany; 9 Department of Human Genetics, Nijmegen Centre for Molecular Life Sciences and Institute for Genetic and Metabolic Disorders, Radboud University Nijmegen Medical Centre, Nijmegen, the Netherlands; 10 University Library and Department of Medical Informatics and Epidemiology, Oregon Health & Sciences University, Portland, OR, USA

## 

Over 100 disease-gene associations have been identified by whole-exome sequencing since the first reports in 2010, leading to a revolution in rare disease-gene discovery [1,2]. However, many cases remain unsolved due to the fact that ~100-1000 loss of function, candidate variants remain after removing those deemed as common, low quality or non-pathogenic. In some cases it may be possible to use multiple affected individuals, linkage data, identity-by-descent inference, identification of *de novo* heterozygous mutations from trio analysis, or prior knowledge of affected pathways to narrow down to the causative variant [3]. Where this is not possible or successful, one approach is to use phenotype data to evaluate whether a particular candidate variant is likely to result in the patient’s clinical manifestations.

Model organism phenotype data represents a highly pertinent but under-utilised resource for such disease gene discovery. Whilst some 1800 human genes were associated with human phenotype ontology annotations (HPO) at the time of publication, a further 5700 genes have been shown to have phenotype data available from mouse and zebrafish model organism databases [4]. We have previously developed algorithmic approaches to semantically compare disease phenotypes with mouse and zebrafish phenotypes for disease candidate gene identification [5-8].

We have previously reported that comparisons to mouse phenotype data can dramatically increase the performance of exome analysis prioritization [9]. In the work presented here we combine the comparison of patient phenotypes to known disease as well as mouse and zebrafish phenotypes for each candidate variant in the exome. Where phenotype data is not available for a candidate we use proximity in protein-protein networks to genes with phenotype data to inform on candidacy based on guilt-by-association. The output is combined with measures of variant candidacy such as pathogenicity and allele frequency and synergistically improves performance: the causative variant is identified as the top hit in up to 96% of exomes for known associations and 49% of exomes for previously undescribed associations.

Our software, Exomiser, is openly available to use at our website [http://www.sanger.ac.uk/resources/databases/exomiser/query] and for download to perform local analysis. We are currently collaborating with the NIH Undiagnosed Disease Program to achieve diagnosis of problematic cases through exome analysis. In conclusion, our results clearly show the value of collecting comprehensive clinical phenotype data for translational bioinformatics and future work will focus on producing a robust solution for clinical diagnostics.
